# Determination of Tetracycline Antibiotics in Milk by Solid-Phase Extraction Using a Coordination Polymer Based on Cobalt Trimesinate as a Sorbent

**DOI:** 10.3390/polym15234539

**Published:** 2023-11-26

**Authors:** Victoria N. Naumkina, Veronika M. Lyamina, Vladimir A. Zhinzhilo, Igor E. Uflyand

**Affiliations:** Department of Chemistry, Southern Federal University, Rostov-on-Don 344090, Russiai06993@yandex.ru (V.A.Z.)

**Keywords:** coordination polymer, trimesic acid, cobalt, adsorption, extraction, tetracycline, milk

## Abstract

The coordination polymer was obtained based on cobalt trimesinate. It was characterized by elemental analysis, IR spectroscopy, X-ray diffraction analysis and scanning electron microscopy. The polymer was studied as a sorbent for solid-phase extraction of tetracycline antibiotics. Cobalt trimesinate had a high adsorption capacity (400 mg/g). Antibiotic adsorption followed the pseudo-second-order kinetic model and the Freundlich isotherm model. The process proceeded spontaneously, as indicated by the calculated thermodynamic parameters. The resulting coordination polymer has good stability and recyclability. The possibility of using cobalt trimesinate for the determination of tetracycline in various milk samples was investigated. This work holds great promise for the development and application of a cobalt trimesinate-based coordination polymer for use in sample preparation to replace the time-consuming vacuum evaporation procedure with a relatively simple solid-phase extraction procedure.

## 1. Introduction

Tetracycline (TC) antibiotics, in particular, oxytetracycline, tetracycline, chlortetracycline, and doxycycline, are antibiotics with a diverse spectrum of action. They are active against Gram-positive and Gram-negative bacteria. TCs are food additives used in dairy farming to prevent or eliminate diseases such as mastitis and metritis in cattle. Antibiotics are included in veterinary drugs due to their low cost, good oral absorption, and a wide variety of properties. Large-scale and illegal use of antibiotic-based drugs in veterinary activities leads to their increase and accumulation in food products of animal origin. TCs are often environmental pollutants, where they enter with wastewater from various pharmaceutical plants, pig farms and poultry farms. The presence of even lesser amounts of TCs in food, water and soil has a negative impact on human health and the environment, causing the development of antibiotic-resistant microorganisms.

To determine TC, liquid chromatography methods with various detection methods are widely used: spectrometric [1,2,3], fluorimetric [4] and mass spectrometric [5]. Enzyme-linked immunosorbent assay (ELISA) is actively used as a test system for determining TC content [6,7]. Chemiluminescent methods are of significant importance for the accurate and sensitive determination of analytes, but they do not allow selective determination of several TCs in a single sample. Electrochemical [8,9] and electrophoretic [10] methods are less common, but they are also used to determine TC.

For the chemiluminescent determination of TC, various types of systems are used in which the analyte directly participates in the chemiluminescent reaction, catalyzes or inhibits it. The chemiluminescent analysis allows for high sensitivity, but it does not allow selective determination of several analytes simultaneously. Many matrix components and salt backgrounds have a significant interfering effect on chemiluminescent reactions; therefore, even after multistage sample preparation, multiple sample dilutions are often required (up to 500 times [11]).

Effective separation of TCs can be achieved using capillary electrophoresis. For quantitative determination, spectrophotometric detection in a capillary is most often used. For more efficient separation, narrow capillaries (<100 µm) are used. However, such a short optical path often cannot provide the required sensitivity. To increase the optical path, the capillary is expanded or bent, which in turn worsens the separation efficiency [12].

For the electrochemical determination of TC, the voltammetry method is used. Using this method, a lower limit of detection (LOD) down to tenths of mg/L can be achieved [8]. The main disadvantage of voltammetry in determining TC is its low selectivity due to the strong overlap of voltammograms. To simultaneously determine several TCs, it has been proposed to use chemometric methods [9].

Currently, in laboratory practice, ELISA-type systems are used to determine TC using enzyme-linked immunosorbent assay [13]. Attempts have been made to develop very specific hapten-based systems. Haptens are low-molecular substances that do not have antigenicity and acquire it with an increase in molecular weight (for example, due to attachment to a special carrier protein) [14]. The main disadvantages of enzyme immunoassays are the high probability of obtaining false-positive and false-negative results, the high cost of commercially available test kits and their limited shelf life.

Reversed-phase liquid chromatography (RPLC) is a widely used method for the efficient chromatographic separation of various compounds, including TCs. The most commonly used columns are 15 and 25 cm in length, with grafted octadecyl or octyl groups. For spectrophotometric detection, mobile phases are used, consisting of solutions of weak organic acids (formic, acetic and oxalic) with a content of up to 0.5% and polar organic solvents (acetonitrile, methanol, less often ethanol, and mixtures thereof). In an acidic environment, the interaction of silanol groups with analytes is reduced, which prevents the formation of asymmetric chromatographic peaks. Ultraviolet detection is most often performed in the wavelength range from 350 to 365 nm. In fluorimetric detection, buffer solutions containing metal ions, most often calcium ions, are used as the mobile phase. The formation of chelate complexes provides more intense fluorescence and, as a result, increases the sensitivity of determination. Detection is carried out at excitation wavelengths of 380–390 nm and emission wavelengths of 490–520 nm [15]. Thus, chromatographic methods of analysis make it possible to selectively determine TC. Important advantages of the methods are low sample consumption and the possibility of automation. Through the implementation of different detection methods, it becomes possible to achieve the required level of sensitivity in the analysis of biological fluids and food products. Due to these advantages, chromatographic methods are most often used to determine the TC content in complex matrices [16].

Another method for determining TC is solid-phase extraction (SPE). This method of separation and concentration involves the sorption of target analytes on the surface of various sorbents (nanomaterials, polymer films, etc.) from the liquid or gas phase, followed by elution or thermal desorption [17,18]. The SPE method is widely in demand due to the possibility of its modification, for example, using coordination polymers (CPs) capable of interacting with antibiotics and forming supramolecular complexes, about which not much is known, and which are capable of luminescence under certain conditions [19]. In addition, the SPE method can be easily automated for fast and on-line analyses since it can be combined with gas or liquid chromatography, photoluminescence [19] and other analytical methods [19,20]. SPE is widely in demand due to the possibility of its automation and ease of combination with gas and liquid chromatography, as well as other analytical methods [20].

Sorption microextraction on the insert of a magnetic stirrer involves the application of a layer of sorbent on the surface of the insert. This approach was applied to extract TCs in the work of [21]. The disk was made of a composite (magnetite coated with copper trimesinate) capable of rotation in a magnetic field. The sorption time was 120 min, and the extraction efficiency was 82–93% for various analytes.

One cost-effective alternative to expensive SPE cartridges is the use of packed SPE cartridges. In this method, a specified amount of sorbent is carefully placed inside a cartridge, and then a liquid sample is passed through a layer of sorbent, allowing it to adsorb and retain the target analytes. Then, elution is carried out with available solvents.

Dispersive SPE involves the addition of a sorbent to the sample, intensive mixing of the suspension, subsequent separation of the sorbent by centrifugation, and elution [20].

Magnetic dispersive SPE is a modification of the dispersive SPE method which uses materials with magnetic properties. This makes it possible to avoid the centrifugation step and separate the volume of the sorbent using an external magnetic field [22].

Due to the low TC content in these matrices and the complexity of their composition, the determination of these compounds requires mandatory sample preparation, which has recently been often carried out using SPE. Problems arising in the SPE process are associated with the significant hydrophilicity of TCs and their tendency to form complexes with metal ions. These factors contribute to the low recovery of these compounds on traditional sorbents. Therefore, it becomes crucial to explore alternative sorbent materials that can quantitatively isolate TCs from various sources.

CPs are crystalline porous materials formed by coordinating organic linkers with metal ions. These complex three-dimensional structures have a number of unique properties, which include high thermal and chemical stability, as well as photocatalytic activity [23]. The presence of intramolecular cavities provides such structures with a large internal surface, and this, in turn, promises them good prospects as effective sorbents for various applications, such as environmental remediation or the removal of contaminants from aqueous solutions. CPs have a uniform cavity structure, constant nanoporosity, high stability and significant surface area, as well as intrapore functionality and external surface modification. Various adsorbents were used for TC adsorption [22,23,24,25,26,27,28,29]. However, many adsorbents have faced problems of instability and low removal efficiency, ultimately reducing their practical use. Therefore, the pursuit of developing more efficient nanomaterials with a higher specific surface area and porous structure has emerged as a potential solution for antibiotic removal. Various methods have been proposed for the removal of antibiotics using CP materials as a new class of adsorbents.

The purpose of this work was to synthesize and use CP (cobalt trimesinate) as a sorbent for SPE of TC from milk. To achieve this goal, the following tasks were conducted: the development of a simple and environmentally friendly method for the synthesis of cobalt trimesinate, the study of the SPE process of TC by cobalt trimesinate from aqueous media, the determination of the kinetic and thermodynamic characteristics of the SPE process and the determination of the TC content in real objects (milk from different manufacturers) using the synthesized sorbent as a reagent in the process of sample preparation (extraction of TC from milk whey into the solid phase).

## 2. Materials and Methods

### 2.1. Materials

Commercially available reagents were used in the work: trimesic acid (98%, Acros Organics, Geel, Belgium), KOH (technical, JSC Caustic, Volgograd, Russia), and CoSO_4_·6H_2_O (99%, Acros Organics, Geel, Belgium). Ethanol, methanol, ethyl acetate, chloroform and diethyl ether were used as solvents (all of the above reagents were produced by JSC Reakhim LLC, Moscow, Russia, and were chemically pure). All reagents were used without preliminary preparation and purification. Solvents were prepared in accordance with known recommendations.

### 2.2. Synthesis of Cobalt Trimesinate

The synthesis of cobalt trimesinate was carried out in two stages. At the initial stage, a soluble form of potassium trimesinate was obtained. A solution containing 0.09 mol of KOH in 50 mL of water was heated to 80 °C, and then 0.03 mol of acid was added. The pH of the solution was adjusted within the range of 5.5–6.0. Moving on to the second stage, an aqueous solution containing 0.09 mol of CoSO_4_·6H_2_O (calculated as an anhydrous salt) in 30 mL of hot water was introduced into the previously prepared solution. The cobalt trimesinate crystals were dried in air until a crumbly dry state was achieved. The product was treated with dry ethanol, ethyl acetate and chloroform. Each solvent was continuously stirred on a magnetic stirrer for 12 h, ensuring thorough purification. The cobalt trimesinate was then air-dried and placed in a dynamic vacuum at 150 °C for 8 h. The yield of the anhydrous product in the form of purple crystals was 88%. Table 1 presents the results of elemental analysis of the obtained cobalt trimesinate.

### 2.3. Characterization

A CHNOS Vario EL cubic analyzer (Elementar Analysensysteme GmbH, Langenselbold, Germany) was used for elemental analysis. Cobalt was determined using AAS3 equipment (VEB Feinmesszeug Fabrik, Zeiss, Germany). X-ray diffraction analysis (XRD) was performed on a Phywe XR 4.0 device (PHYWE Systeme GmbH & Co. KG, Göttingen, Germany, CuKα, λ = 0.15418 nm, scanning speed 2 deg/min, step size 0.02°). Fourier transform infrared (FTIR) spectra were obtained on a Perkin-Elmer Spectrum 100 FTIR spectrometer (PerkinElmer, Waltham, MA, USA) using KBr pellets and Softspectra data analysis software (Shelton, CT). Scanning electron microscopy (SEM) was performed on a ZEISS Crossbeam 340 instrument (Carl Zeiss AG, Jena, Germany) at an accelerating voltage of 3 kV. Secondary electrons were detected using an Everhart–Thornley detector (SE2), with magnification varying from 1.92 to 50,000 times.

### 2.4. Tetracycline Adsorption Study

To evaluate the adsorption and kinetics of the tested solution of TC, a batch method was used [30]. The experimental part consisted of placing a TC solution with a concentration of 100 mg L^−1^ and a volume of 200 mL, thermostated at 10, 20 and 35 °C, on a magnetic stirrer. The rotation speed of the stirrer was carefully controlled to ensure efficient mixing without introducing air into the liquid phase. When the solution reached the required temperature, to initiate the adsorption process, 20 mg of sorbent was added. Every 15 min, a 10 mL sample of the sorbent suspension was taken and subjected to centrifugation. The resulting filtrate was then analyzed spectrophotometrically to determine the concentration of residual TC at λ_max_ = 420 nm. In addition, the adsorption behavior was studied under various conditions, including changes in the pH of the medium, the initial concentration of TC and the dosage of the sorbent. These parameters were systematically varied to evaluate their impact on the adsorption process.

The adsorption value was calculated using the following equations:

q_t_ = (C_0_ − C_t_)V/m (1)

q_e_ = (C_0_ − C_e_)V/m (2)

where q_t_ and q_e_ are the quantities (mg g^−1^) of TC adsorbed on the adsorbent at time t and equilibrium, respectively; C_0_, C_t_ and C_e_ are the TC concentrations in solution (mg L^−1^) initially, at time t and at equilibrium, respectively; m (g) and V (L) represent the amount of adsorbent and the volume of the TC solution, respectively.

The degree of adsorption R (%) (extraction ratio of the sorbate) was calculated by the following formula:

R (%) = (C_0_ − C_e_)/C_0_ × 100% (3)

#### 2.4.1. Dependence of Adsorption on the pH of the Medium

The experiment was conducted under controlled conditions at T = 20 °C. To start the experiment, 200 mL of TC solution and 20 mg of sorbent were mixed in a beaker. The beaker was then placed in a temperature-controlled environment and kept under constant stirring. The samples of 10 mL were extracted from the beaker at predefined time intervals. The pH of the medium was controlled using a pH meter and corrected by adding 0.1 N NaOH or HCl. The collected samples were centrifuged immediately after being placed in centrifuge tubes to exclude the continuation of the extraction process. The concentration of residual TC was determined as described previously.

#### 2.4.2. Dependence of Adsorption on Tetracycline Concentration

The experiment was carried out at 10, 20 and 35 °C. A total of 40 mL of TC solution at concentrations of 100, 50, 25, 12.5 and 6.125 mg L^−1^ was placed in 5 beakers, and 20 mg of the sorbent was added to each. The mixtures were thermostated at a given temperature and stirred on a magnetic stirrer for 3 h, and 10 mL samples were taken. The concentration of residual TC was determined as described previously.

#### 2.4.3. Number of Working Cycles

To do this, a layer of filling material was placed in a column with a diameter of 1.7 cm, then the column was filled with the studied cobalt trimesinate to a depth of 15 cm. To compact the sorbent layer, the bottom of the column was periodically tapped on the table surface. A total of 20 mL of the test solution of TC was poured. After 15 min, elution began. The eluent was a mixture of methanol and hydrochloric acid (4:1). The determination of TC in the eluate was carried out by a qualitative drop reaction with FeCl_3_; the appearance of a blue color indicates the presence of TC. It was established that the beginning of TC elution occurred after the passage of 1 mL of blank eluent. The TC concentration was determined spectrophotometrically. The experiment was carried out 3 times. SPE of TC from whey was carried out in the same way.

#### 2.4.4. Study of Adsorption on a Real Object

Milk from five different producers in different price categories was chosen as a real object: farm milk (sample 1), ultra-pasteurized (sample 2), pasteurized (sample 3), sterilized (sample 4) and baked (sample 5). The determination of TC in milk involves the passage of several successive stages. The first stage of determining TC in milk includes primary sample preparation—the removal of proteins and milk fat from the sample. To do this, 2–3 mL of concentrated acetic acid was added to 250 mL of the analyzed product and thermostated at 40–45 °C for 5–10 min. Next, the sample was mixed and centrifuged for 5–7 min at 3000–5000 rpm. The centrifugate was transferred into a flask, the pH of the medium was adjusted to ≈2.5 with nitric acid (5 M) and 25 mL of a buffer solution (pH = 2.5) was added. At the second stage of sample preparation, the obtained whey was evaporated in a vacuum by about 70–80% to concentrate TC until LOD was reached. We used this technique as a comparison. In the experiment, milk whey was obtained as described earlier without using a long vacuum concentration procedure; 40 mL of whey was taken, and 20 mg of the sorbent was added. The mixture was stirred with a magnetic stirrer for 3 h at room temperature. After mixing, a 10 mL sample was taken and then subjected to centrifugation. The concentration of TC in the resulting centrifugate was determined using spectrophotometric analysis. This analysis was crucial to evaluate the efficiency of the TC extraction process. In addition, the obtained results helped to determine the active sorbent layer present in the column. Following this, the column was assembled and filled with a conditioned sorbent, and studies were carried out as described earlier.

## 3. Results and Discussion

### 3.1. Synthesis and Study of the Sorbent

Currently, several methods for the synthesis of cobalt trimesinate have been developed, but they are labor-intensive and time-consuming. Therefore, we have developed a readily available synthesis method by reacting cobalt nitrate hexahydrate and trimesic acid in water. Cobalt trimesinate was synthesized via direct reaction of a metal salt and trimesic acid in water in the presence of an alkali.

**Figure 1:**
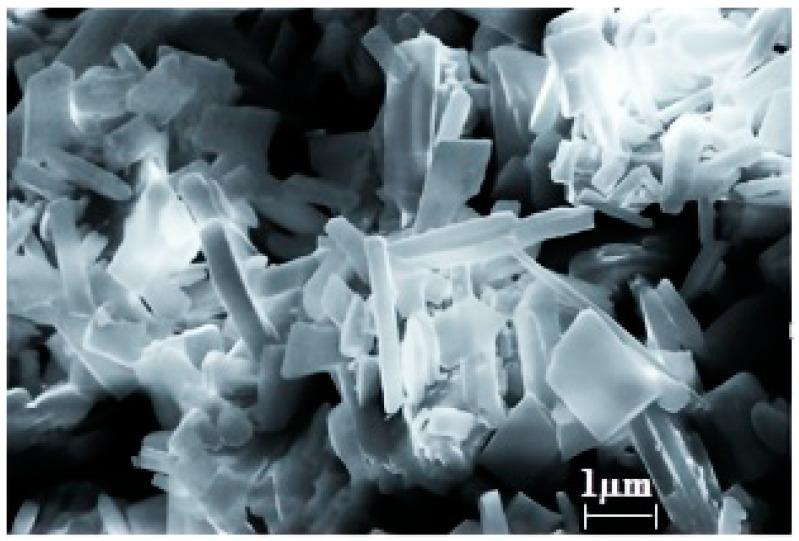
SEM image of cobalt trimesinate crystals.

The morphology of the synthesized cobalt trimesinate was examined using SEM analysis. Cobalt trimesinate is formed by elongated prismatic crystals which are homogeneous and monolithic (with a smooth surface). The size of the crystals varies from 3.1 × 0.6 × 0.6 (15%) to 3.6 × 0.5 × 0.5 (80%) µm and less than 2% is represented by wide but short crystals (in the form of tablets) with dimensions of 2.6 × 1.86 × 0.33 µm (Figure 1).

**Figure 2:**
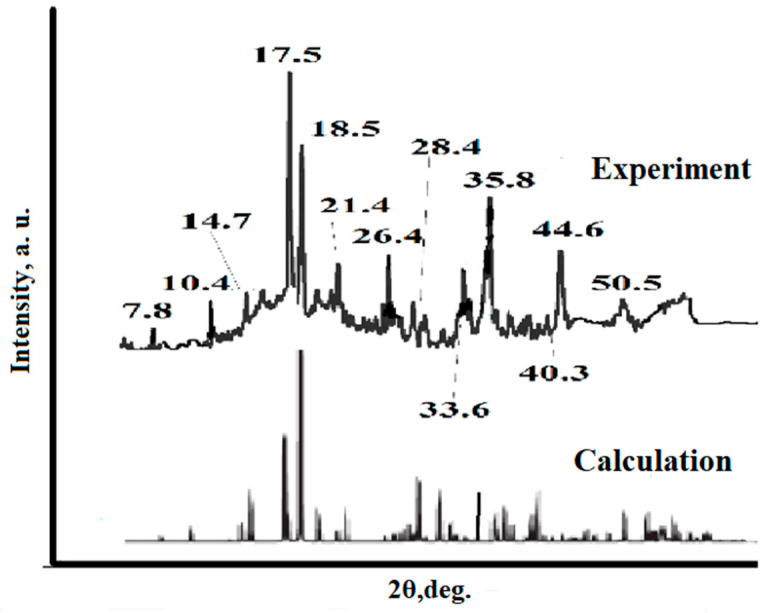
Calculated experimental diffraction patterns of cobalt trimesinate.

To identify the substance synthesized by us, XRD analysis was carried out. According to the results of the analysis, distinct peaks are visualized on the diffraction curve which indicate that the obtained sample has a good degree of crystallinity and is characterized by phase purity. The reflection angles of the diffraction peaks are in fairly accurate agreement with the simulation results performed in the MatH-3 X-ray diffraction data processing program (Figure 2). In addition, they coincide with the results of previously published works [31,32]. An analysis of the experimental curve and simulation reflections shows that the relative intensity of the peaks obtained in the experiment does not fully correspond to the theoretical calculation. The latter circumstance with a high degree of probability can be interpreted as evidence of the existence of crystals with a predominant growth orientation and also characterizes the crystals of the compound as being in the stage of formation.

**Figure 3:**
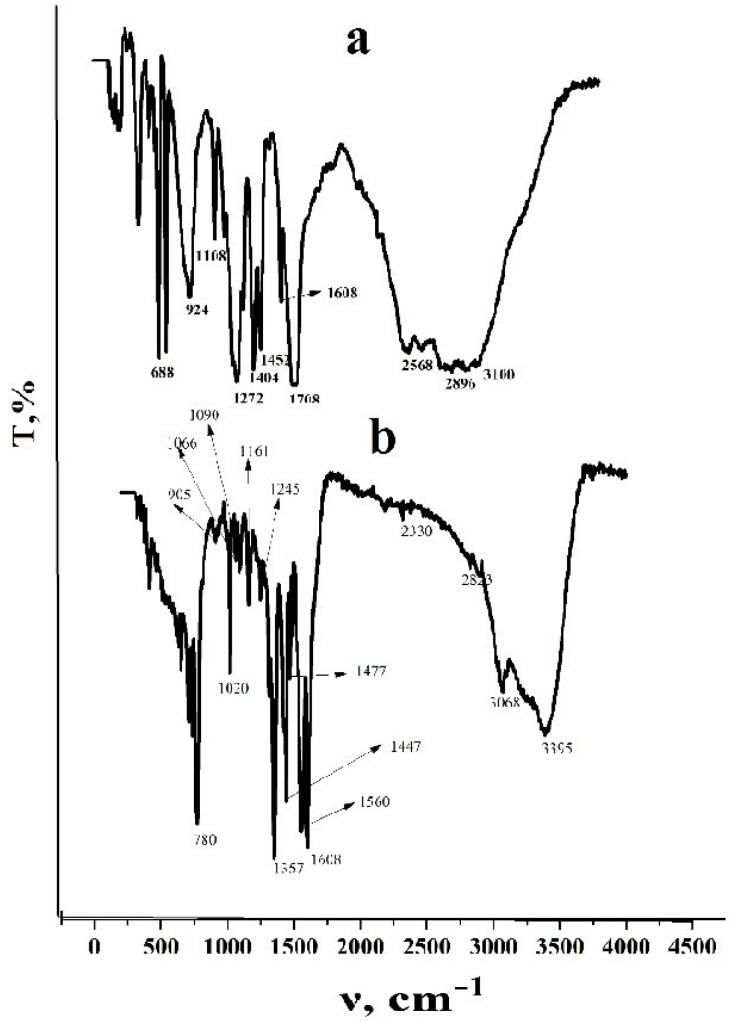
IR spectra of cobalt trimesinate (**a**) and trimesic acid (**b**).

To determine the possible mode of coordination of the trimesinate ligand, the IR spectra of trimesic acid and the resulting coordination polymer are studied (Figure 3). In the IR spectrum of the starting acid, bands with an absorption maximum in the region of 1720, 1430 and 1276 cm^−1^ are recorded. The first absorption peak is characteristic of stretching vibrations of the carboxyl group, and the other two are explained by a combination of planar bending vibrations of hydroxo groups and stretching vibrations of the C–O bond in dibasic carboxylic acid molecules. The obtained spectra of cobalt trimesinate are consistent with the previously published results [31,32]. Characteristically, the IR spectrum of the resulting coordination polymer does not contain a band at 1720 cm^−1^, which indicates complete deprotonation of carboxyl groups and their binding to metal ions. The latter circumstance corresponds to the fact of the formation of an M-O bond and is illustrated by the formation of a strong absorption band in the low-frequency vibration region at 760 cm^−1^. In this case, absorption bands appear that were previously absent in the precursor in the region of 1560 cm^−1^ which are characteristic of asymmetric stretching vibrations of carboxylate ions. In addition, the appearance of symmetric stretching vibrations of this anion in the region of 1375 cm^−1^ should be noted. The difference in the wave numbers of the asymmetric and symmetric stretching vibrations of carboxylate anions is less than 185 cm^−1^, which allows us to conclude that the formation of a bidentate-bridge mode of coordination of carboxylate ions is possible.

### 3.2. Solid-Phase Extraction of Tetracycline

**Figure 4:**
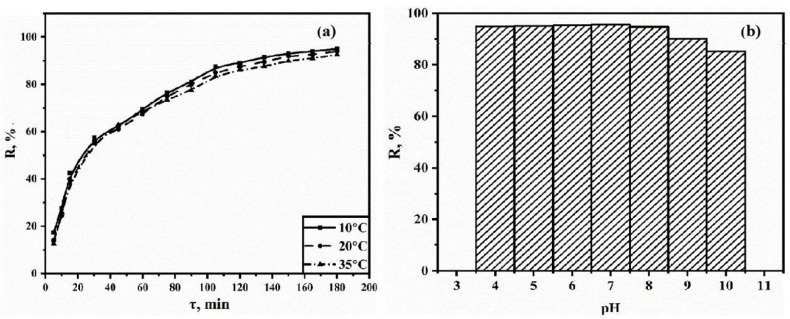
Dependence of the degree of adsorption on the contact time at different temperatures (**a**) and dependence of the degree of adsorption on the pH of the medium (**b**).

The adsorption capacity of cobalt trimesinate toward TC in aqueous solutions was investigated. Figure 4a illustrates the relationship between the degree of adsorption and the time at different temperatures. Analysis of the data shows that the degree of adsorption is higher at lower temperatures. However, it is noteworthy that the degree of adsorption remains above 90% for all three temperature conditions, indicating satisfactory adsorption performance at each temperature. In addition, the analysis of TC sorption isotherms shows that they relate to monomolecular sorption isotherms and the curves monotonically approach a certain limiting value corresponding to filling the monolayer.

The relationship between the degree of adsorption and the pH of the medium is shown in Figure 4b. The data illustrate that the degree of adsorption is higher in acidic and neutral media and decreases in alkaline media.

**Figure 5:**
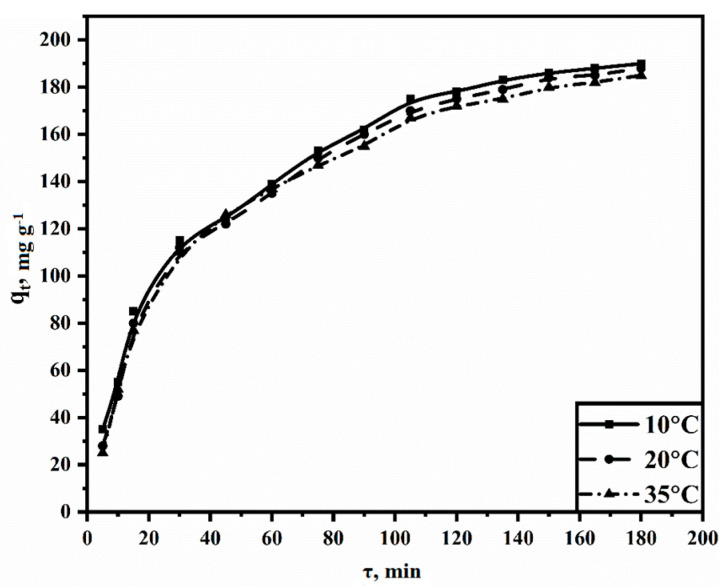
Kinetic curves of solid-phase extraction versus contact time at various temperatures.

Figure 5 illustrates that most of the sorbate is adsorbed within the first 90 min, followed by a gradual leveling off and reaching a plateau after approximately 160–180 min. This trend can be explained by the initial abundance of free sorption sites that become filled during the process. In general, we can conclude that equilibrium is achieved approximately 3 h after the start of the interaction.

**Figure 6:**
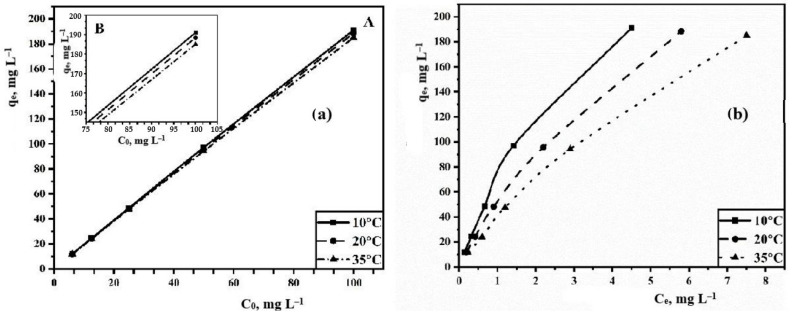
(**a**) Dependence of the TC sorption on the initial concentration of TC at various temperatures. A—full-scale plot; B—scaled plot. (**b**) Dependence of the TC sorption on the equilibrium concentration of TC.

Figure 6a shows the dependence of TC sorption on its initial concentration. The data obtained during the experiment indicate that the sorption capacity increases with increasing initial sorbate concentration. The graph reaches a plateau at the optimal concentration of 100 mg L^−1^. Consequently, the sorbent is capable of adsorbing a large amount of antibiotic, exceeding the maximum permissible concentration (MPC) set at 100 mg L^−1^. With an increase in temperature, the sorption capacity decreases and the general trend persists. With increasing temperature, the sorption capacity decreases, but the general trend remains the same.

The dependences of the maximum sorption capacity on the equilibrium concentration of TC are shown in Figure 6b. At lower concentrations, there is a steep and significant increase in the degree of adsorption. Further, it gradually becomes gentle with the increasing concentration. This pattern indicates that the sorption process uses the maximum number of available active sorption centers. As the sorbate concentration increases, the number of active sites gradually decreases.

Summarizing the obtained dependences, we can determine the most favorable parameters for the adsorption of TC by cobalt trimesinate. The initial concentration of the pollutant varies from 6.125 to 100 mg L^−1^. In this case, the best sorption capacity is achieved at an initial dye concentration of 6.125 mg L^−1^. The optimal weight of the sorbent is 20 mg. According to kinetics, equilibrium occurs 3 h after the introduction of the coordination polymer. Low temperatures are most favorable for sorption. The most favorable pH ranges from strongly acidic to neutral.

### 3.3. Adsorption Isotherms

Experimental data on the adsorption of TCs by the studied coordination polymer were analyzed using the Langmuir and Freundlich adsorption models.

#### 3.3.1. Langmuir Adsorption Model

This model provides a reliable quantitative description of the adsorption process, accurately representing both low and high sorbate concentrations. According to the Langmuir adsorption isotherm, adsorption takes place at specific homogeneous sites within the sorbent and on its surface. The Langmuir model is widely used and has proven to be effective for predicting monolayer adsorption processes in various applications [33]. The Langmuir isotherm can be written as follows [34]:

(4) $$q_{e}=\frac{q_{max}K_{L}C_{e}}{1+K_{L}C_{e}}$$

where K_L_ is the Langmuir constant (mg L^−1^) related to the set of binding sites and the free energy of sorption; q_max_ is the adsorption capacity expressing the concentration of TC during the formation of a monolayer on the sorbent (mg g^−1^).

Equation (4) is usually represented in a linear form:

(5) $$\frac{C_{e}}{q_{e}}=\frac{1}{K_{L}q_{max}}+\frac{C_{e}}{q_{m}}$$

**Figure 7:**
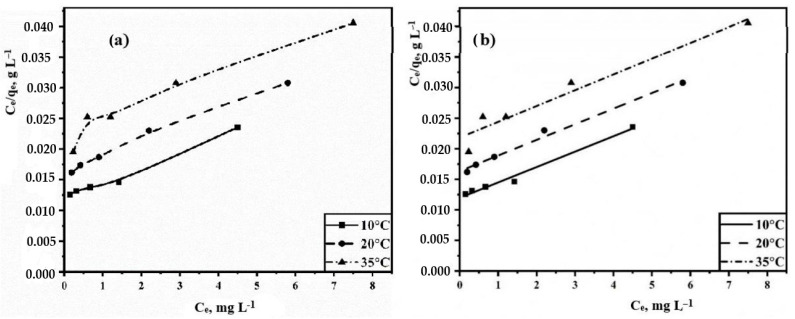
Langmuir isotherms of solid-phase extraction of TC in non-linearized (**a**) and linearized (**b**) forms.

As a result of the approximation of Equation (5), the thermodynamic constants were obtained based on empirical data (Table 1). Plots of Langmuir isotherms in non-linearized (Figure 7a) and linearized (Figure 7b) forms, as well as approximation equations are presented. The shape of the isotherms indicates a clear dependence of the adsorption efficiency: a decrease in temperature leads to an increase in the rate of sorbate absorption. The calculated values of the maximum monolayer capacity and sorption equilibrium constants at different temperatures have a clear pattern: q_max_ (10 °C) > q_max_ (20 °C) > q_max_ (35 °C); K_L_ (10 °C) > K_L_ (20 °C) > K_L_ (35 °C). This once again indicates a preference for low temperatures.

#### 3.3.2. Freundlich Adsorption Model

The Freundlich adsorption isotherm model is usually used to describe heterogeneous systems with average degree of coverage of the sorbent surface. It is presented below:

(6) qe=kFCe1nF

where K_F_ is a constant that expresses the adsorption capacity of the adsorbent (mg^1−1/n^ L^1/n^ g^−1^); n is an empirical constant which is related to the magnitude of the driving force of adsorption.

The value 1/n quantitatively characterizes the degree of surface inhomogeneity of the coordination polymer [35].

**Figure 8:**
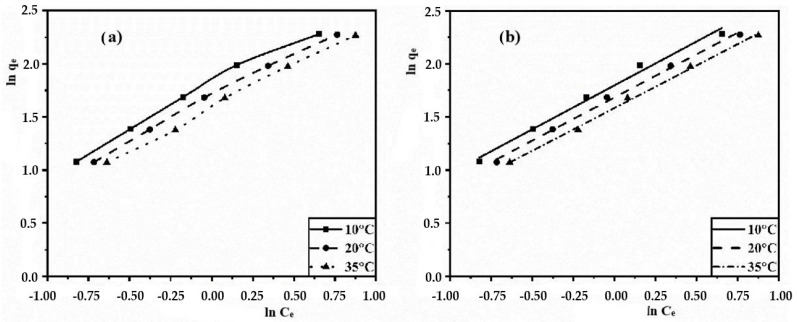
Freundlich isotherms in non-linearized (**a**) and linearized (**b**) forms.

By applying Equation (6) to the experimental data, Freundlich isotherms were obtained in non-linearized (Figure 8a) and linearized (Figure 8b) forms, approximation equations and constants (Table 2).

When comparing the values of the correlation coefficient (R^2^), we can conclude that the Freundlich model describes the adsorption process in more detail. Notably, the coefficient values (1/n) range from 0 to 1, indicating favorable adsorption conditions. In addition, it is observed that the highest values of the coefficient are associated with decreasing temperature, which is in good agreement with the principles of the Langmuir model.

Table 3 presents a comparative description of the sorption capacity of different sorbents.

#### 3.3.3. Adsorption Kinetics

The results of adsorption studies are considered using a variety of kinetic models that make it possible to characterize the processes under study as pseudo-first and pseudo-second order reactions. Lagergren proposed an equation that effectively describes solid-phase extraction and can be expressed differentially as follows [40]:

(7) $$\frac{dq_{t}}{dt}=k_{1}(q_{e}-q_{t})$$

The differential form of the classical pseudo-second-order rate equation is as follows:

(8) $$\frac{{dq}_{t}}{dt}=k_{2}({q_{e}-q_{t})}^{2}$$

or

(9) $$\frac{1}{q_{t}}=\frac{1}{k_{2}q_{e}^{2}}+\frac{1}{q_{e}}t$$

transforming which you obtain the following:

(10) $$q_{t}=\frac{t}{\frac{1}{k_{2}q_{e}^{2}}+\frac{t}{q_{e}}}$$

**Figure 9:**
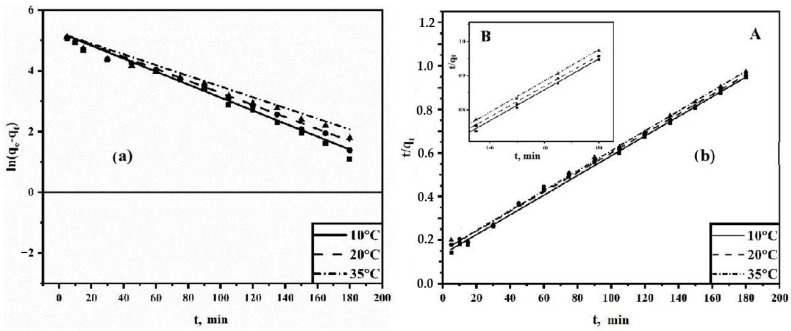
(**a**) Pseudo-first order extraction rate model; (**b**) pseudo-second order extraction rate model. A is a full-scale plot; B is a scaled plot.

For TC adsorption, the kinetic parameters were determined by plotting direct graphs of ln(q_e_ − q_t_) versus t for a pseudo-first order reaction and t/q_t_ versus t for a pseudo-second order reaction (Figure 9a,b).

From the obtained graphs, the kinetic parameters were determined under various experimental conditions and are presented in Table 4. The pseudo-first order equation satisfactorily describes the adsorption patterns observed at the initial stages of the process, especially when film diffusion becomes decisive and has a significant impact on the overall dynamics of the process. Apparently, at the initial stages of the process with low occupancy of the adsorption space, an increase in the concentration of sorbate molecules on the surface of the sorbent leads to their migration into the pores of the sorbent due to the concentration gradient. However, over time, this process slows down noticeably, thereby facilitating the emergence of alternative mechanisms. Based on the calculated values of the coefficient of determination, it can be concluded that the pseudo-first order kinetic model describes the adsorption process less accurately. Therefore, under these conditions, the pseudo-second order kinetic model is considered preferable.

The pseudo-second-order kinetic model assumes that the rate-limiting step is chemical sorption or chemisorption and predicts the behavior over the whole range of adsorption. In this condition, the adsorption rate is dependent on adsorption capacity and not on the concentration of the adsorbate. One major advantage of this model is that the equilibrium adsorption capacity can be calculated from the model; therefore, there is theoretically no need to evaluate the adsorption equilibrium capacity from the experiment.

### 3.4. Thermodynamics of Adsorption

To establish the possibility of a spontaneous adsorption process and to verify the obtained experimental data, we used three main thermodynamic parameters: the change in enthalpy (ΔH^0^), entropy (ΔS^0^) and Gibbs free energy (ΔG^0^). The effect of temperature on equilibrium adsorption was evaluated using the Gibbs equation:

(11) $$∆G^{0}=-RTlnK_{c}$$

where K_c_ is the thermodynamic equilibrium constant, which can be determined from the following equation:

(12) $$K_{c}=\frac{C_{t}}{C_{e}}$$

Considering changes in concentrations in real time and depending on the weight of the sorbent, Equation (12) can be transformed into the following form:

(13) $$K_{c}=\frac{\left(C_{0}-C_{e}\right)V}{mC_{e}}$$

The Gibbs equation for describing specific conditions can also be written as follows:

(14) $$∆G^{0}=∆H^{0}-T∆S^{0}$$

Combining the equations above, we obtain the following equation:

(15) $${lnK}_{c}=\frac{∆S^{0}}{R}-\frac{∆H^{0}}{RT}$$

**Figure 10:**
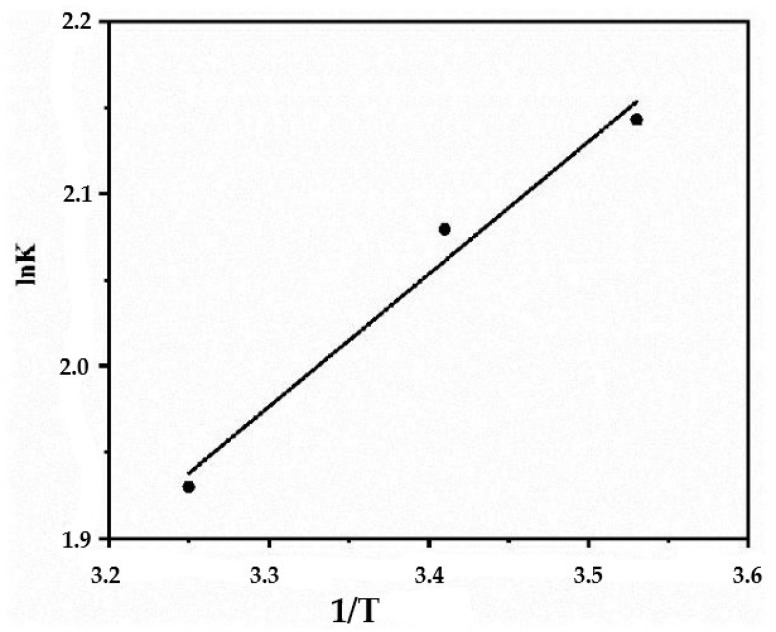
Dependence of the logarithm of the thermodynamic constant on the reciprocal temperature.

The thermodynamic parameters of the process were determined by graphical calculation (Figure 10) based on the relationship between the thermodynamic equilibrium constant and the inverse temperature. The obtained values are presented in Table 5.

From the data given in Table 4, it can be seen that the adsorption process is spontaneous. Temperature-dependent Gibbs free energy values indicate increased adsorption efficiency at moderately low temperatures. The negative enthalpy of adsorption confirms the exothermic nature of the process. In addition, a positive entropy value indicates an increase in the number of degrees of freedom at the solid–liquid interface during the adsorption process of TC. This reflects the affinity of the sorbate for the sorbent molecules.

### 3.5. Number of Extraction Cycles after Regeneration

**Figure 11:**
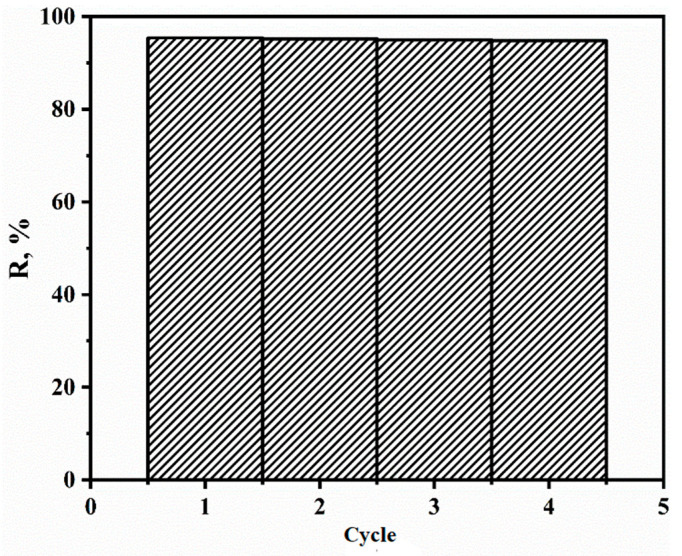
Dependence of the degree of extraction of TC on the number of working cycles.

After an experiment on sorbent regeneration using an eluent (Figure 11), it was found that this sorbent can be used four times.

**Figure 12:**
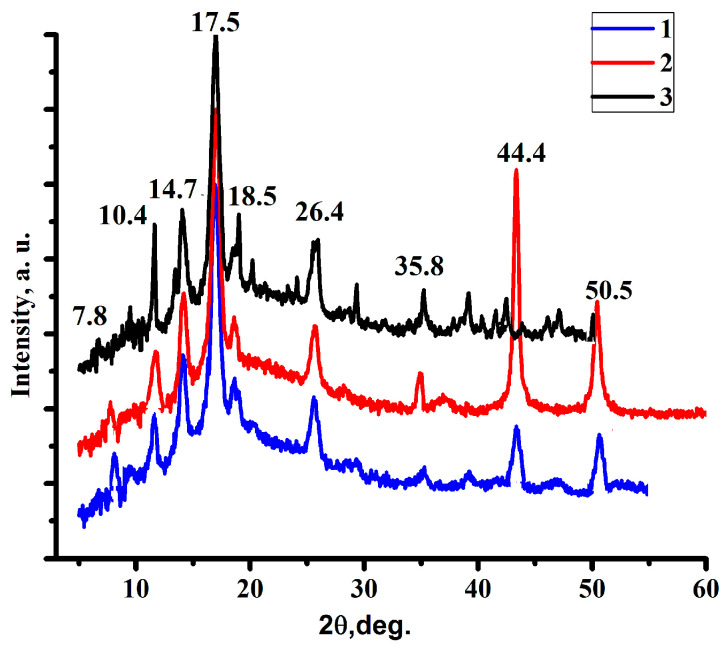
XRD spectra of the coordination polymer obtained after desorption of TC. 1—pH = 4, 35 °C; 2—pH = 7, 35 °C; 3—pH = 10, 35 °C.

The stability of the coordination polymer after desorption of TC and its activation at different pH values of the medium can be proven by the XRD curves presented in Figure 12.

### 3.6. Determination of the Sorption Activity of CP on Real Objects

The object of this study was milk from five different manufacturers of different price categories and different methods of heat treatment: purchased from a farmer (sample 1), purchased from a supermarket—ultra-pasteurized (sample 2), pasteurized (sample 3), sterilized (sample 4 and, melted (sample 5). During a preliminary study of purchased milk, an antibiotic belonging to the TC group was isolated, which we identified as tetracycline hydrochloride (7-chloro-4-dimethylamino-1,2,3,4,4α5,5α,6,12,12α-decahydro-6,10,11,12α-tetraoxy-6-methyl-1,3,12-trioxo-2-naphthacenecarboxamide hydrochloride). After precipitation of proteins and fats in the manner described earlier, the resulting whey was divided into two parts. The first part was additionally prepared according to a previously described procedure: it was evaporated in a vacuum at 30 °C to a residual volume of 30% of the initial serum volume, then examined on a spectrophotometer at a wavelength of 420 nm and compared with a series of standard solutions of TC. The second part of the serum was treated with a sorbent at a dose of 100 mg per 200 mL of serum and was continuously stirred on a magnetic stirrer for 45 min at 20 °C. The suspension was allowed to settle for a brief time and subjected to centrifugation. The centrifugate was discarded, and TC was eluted from the precipitate with acidified methanol as described earlier, and the amount of TC content in the eluate was determined spectrophotometrically at a wavelength of 420 nm, also compared with a series of standard solutions.

When processing the data, the following metrological characteristics were considered:

1. Repeatability index (σ_r_);
2. Intra-laboratory reproducibility (σ_R_);
3. Accuracy index (±Δ_c_);
4. Accuracy rate (±Δ).

The summarized results are presented in Table 6.

Of the five studied objects, TCs were detected only in three (samples 1, 2 and 3). The results of determining TC in the studied food products are presented in Table 7.

**Figure 13:**
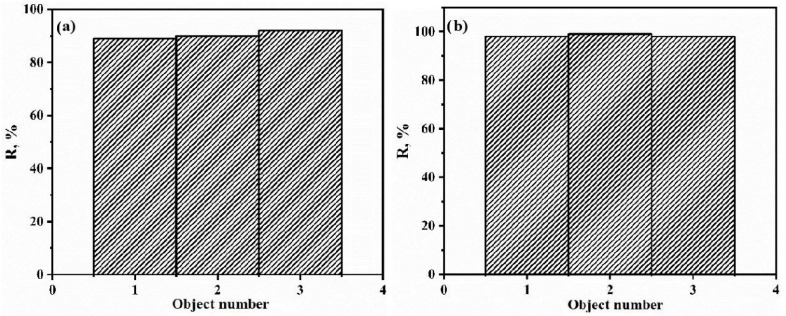
(**a**) The degree of adsorption of TC in real objects; (**b**) the degree of adsorption of TC after passing through the sorbent layer.

Next, adsorption was carried out at 20 °C with the addition of 20 mg of the sorbent after stirring for 3 h. The remaining concentration of TC in the serum was determined using a spectrophotometer at λ_max_ = 420 nm. The data obtained during the experiment are presented in Figure 13a.

To check the removal of TC from the milk sample without evaporation, the whey was passed through a column with a layer of sorbent. To do this, a small layer of cotton wool and silica gel was placed in a column with a diameter of 1.7 cm, then the column was filled with cobalt trimesinate while being well tamped. When the layer length was 7 cm, 20 mL of the test solution of TC was poured in. After the solution passed through the sorbent layer, a 10 mL sample was taken. Then, the concentration of the remaining TC was measured using a spectrophotometer at λ_max_ = 420 nm.

The data obtained during the experiment are presented in Table 8. From the obtained data, it can be seen that the degree of extraction is close to 100% (Figure 12), which is a particularly good result.

Based on empirical data, it can be concluded that the determination of TC using SPE gives better results than the determination using whey evaporation.

## 4. Conclusions

In this study, a «green» method for the synthesis of cobalt trimesinate in an alkaline aqueous medium is presented. In addition, the potential application of cobalt trimesinate as a sorbent for solid-phase extraction of tetracycline from aqueous solutions was investigated. The equilibrium adsorption data were collected and analyzed using both the Langmuir and Freundlich adsorption models at temperatures ranging from 10 to 35 °C. The obtained values of the correlation coefficients allow us to conclude that the process of tetracycline adsorption on the surface of cobalt trimesinate is most accurately described by the Freundlich isotherm equation. The coefficient 1/n ranges from 0 to 1, indicating a favorable adsorption process. Experimental determination of thermodynamic parameters (ΔG^0^, ΔH^0^, ΔS^0^) confirms the idea that tetracycline adsorption is a spontaneous process of a weakly exothermic nature. Since the ΔG^0^ index has a calculated value of up to 40 kJ/mol, it can be argued that the adsorption process is a predominantly physical nature—physical sorption. The sorbent was found to have a high adsorption degree of over 95% within four cycles. When working with real objects, the sorbent under study showed satisfactory results for the adsorption of tetracycline under normal conditions, which can be used in sample preparation during analysis, and it is possible to replace the lengthy vacuum evaporation procedure with a relatively simple solid-phase extraction procedure.

## Figures and Tables

**Table 1:** Elemental analysis of synthesized cobalt trimesinate ^1^.

Element	Found, %	Calculated for Co_3_(L)_2_, %
C	36.8	36.56
H	0.99	1.01
Co	30.2	29.92

^1^ Co_3_(L)_2_ is cobalt trimesinate, L is C_6_H_3_(COO)_3_^3–^.

**Table 2:** Parameter values of adsorption isotherms.

Model	T, °C		
	10	20	35
Langmuir	q_max_ = 400.25 mg g^−1^ K_L_ = 0.25 L mg^−1^ R^2^ = 0.956	q_max_ = 391.53 mg g^−1^ K_L_ = 0.1562 L mg^−1^ R^2^ = 0.978	q_max_ = 386.92 mg g^−1^ K_L_ = 0.1184 L mg^−1^ R^2^ = 0.946
Freundlich	1/n = 0.811 K_F_ = 62.74 R^2^ = 0.995	1/n = 0.813 K_F_ = 48.47 R^2^ = 0.996	1/n = 0.804 K_F_ = 38.37 R^2^ = 0.997

**Table 3:** Comparison of the results obtained in this study with previously published studies.

Adsorbent	q_max_, mg g^−1^	References
cobalt trimesinate	400.25	this work
zirconium terephthalate	139.40	[27]
BMMIPs * based on Fe_3_O_4_	236.40	[36]
MOF 818	442.5	[37]
palm-based biochar	76.92	[38]
carbon nanotubes	269.25	[38]
carbon nanotubes	210.43	[39]

* BMMIPs—biocompatible magnetic molecularly imprinted polymers.

**Table 4:** Kinetic parameters of the adsorption process.

Model	T, °C		
	10	20	35
	q_e_ = 193.10 mg g^−1^ k_1_ = 0.02128 min^−1^ k_2_ = 0.00020	q_e_ = 192.80 mg g^−1^ k_1_ = 0.01907 min^−1^ k_2_ = 0.00018	q_e_ = 191.20 mg g^−1^ k_1_ = 0.01753 min^−1^ k_2_ = 0.00018
Pseudo-first order	R^2^ = 0.9633	R^2^ = 0.9604	R^2^ = 0.9589
Pseudo-second order	R^2^ = 0.9901	R^2^ = 0.9973	R^2^ = 0.9976

**Table 5:** Thermodynamic parameters of TC adsorption in the temperature range 10–35 °C.

T, °C	K_c_	ΔG^0^, kJ mol^−1^	ΔH^0^, kJ mol^−1^	ΔS^0^, J mol^−1^ K^−1^
283	8.53	−5.042	−6.41	4.72
293	8.08	−5.065		
308	6.89	−4.941		

**Table 6:** Metrological characteristics of the spectrophotometric method for the determination of tetracycline.

Metrological Characteristics	Determination with Whey Evaporation	Determination Using SPE
LOD	0.3 mg kg^−1^	0.1 mg kg^−1^
R^2^	0.917	0.985
Sample variance of the results of parallel determinations, S^2^	4·10^–7^	1.1·10^–6^
Repeatability index σ_r_, %	91 ± 4%	92.4 ± 2%
*Intra-laboratory* reproducibility σ_R_, mg kg^−1^	0.0074 ± 0.00578	0.0062 ± 0.00274
Accuracy index ±Δc, % (systematic error)	3.4 ± 5%	3.1 ± 2%
Accuracy rate ±Δ, % (error characteristic)	5.2 ± 3%	4.7 ± 2%

**Table 7:** Results of determining the content of TC in milk via SPE with whey evaporation.

Object of Study	TC Concentration before SPE, mg kg^−1^	TC Concentration after SPE, mg kg^−1^	Degree of Extraction of TC, %
Farm (sample 1)	0.0467	0.00051	92
Ultra-pasteurized (sample 2)	0.0305	0.00035	90
Pasteurized (sample 3)	0.0237	0.00089	89

**Table 8:** Results of determining the content of TC in milk via SPE without whey evaporation.

Object of Study	TC Concentration before SPE, mg kg^−1^	TC Concentration after SPE, mg kg^−1^	Degree of Extraction of TC, %
Farm (sample 1)	0.0467	0.00093	98
Ultra-pasteurized (sample 2)	0.0305	0.00031	99
Pasteurized (sample 3)	0.0237	0.00047	98

## Data Availability

Data are contained within the article.
